# Prevalence and clinical presentation of *Rickettsia*, *Coxiella, Leptospira, Bartonella* and chikungunya virus infections among hospital-based febrile patients from December 2008 to November 2009 in Bangladesh

**DOI:** 10.1186/s12879-017-2239-6

**Published:** 2017-02-13

**Authors:** Labib Imran Faruque, Rashid Uz Zaman, Emily S. Gurley, Robert F. Massung, A. S. M. Alamgir, Renee L. Galloway, Ann M. Powers, Ying Bai, Michael Kosoy, William L. Nicholson, Mahmudur Rahman, Stephen P. Luby

**Affiliations:** 1 International Centre for Diarrheal Diseases Research, Bangladesh (icddr,b), Dhaka, Bangladesh; 2 Centers for Disease Control and Prevention (CDC), Atlanta, GA USA; 3 Institute of Epidemiology, Disease Control and Research (IEDCR), Dhaka, Bangladesh

**Keywords:** Febrile illness, *Rickettsia*, *Coxiella*, *Leptospira*, *Bartonella*, Chikungunya virus, Bangladesh

## Abstract

**Background:**

We conducted a study to identify *Rickettsia, Coxiella, Leptospira, Bartonella,* and Chikungunya virus infections among febrile patients presenting at hospitals in Bangladesh.

**Methods:**

We collected blood samples from patients at six tertiary hospitals from December 2008 to November 2009 and performed laboratory tests at the United States Centers for Disease Control and Prevention (CDC).

**Results:**

Out of 720 enrolled patients, 263 (37%) were infected with *Rickettsia;* 132 patients had immunofluorescence antibody titer >64 against spotted fever, 63 patients against scrub typhus fever and 10 patients against typhus fever. Ten patients were identified with *Coxiella*. We isolated *Leptospira* from two patients and *Bartonella* from one patient. Ten patients had antibodies against Chikungunya virus. The proportion of patients who died was higher with rickettsial fever (5%) compared to those without a diagnosis of rickettsial infection (2%). None of the patients were initially diagnosed with rickettsial fever.

**Conclusions:**

Rickettsial infections are frequent yet under-recognized cause of febrile illness in Bangladesh. Clinical guidelines should be revised so that local clinicians can diagnose rickettsial infections and provide appropriate drug treatment.

## Background

In Bangladesh, dengue, malaria, typhoid fever and influenza like illnesses are common causes of febrile illness [1–4]. Other agents might be important causes of fever but are rarely considered by clinicians and often under-diagnosed.

Rickettsial pathogens are gram-negative bacteria causing fever and rash, usually transmitted to humans by contamination of bite sites or skin abrasions with *Rickettsia-*containing flea feces or directly by the bite of ticks [5]. *C. burnetii*, an obligate intracellular and small gram-negative bacterium, has been classified in the Rickettsiales order due to its recovery from ticks and its inability to grow in axenic medium. However, according to recent investigations based on 16S rRNA sequence, *Coxiella* genus belongs to the gamma subdivision of Proteobacteria and *Rickettsia* genus to the alpha-1 subgroup of Proteobacteria [6]. Recently 40 rickettsial cases were detected in a hospital based study in Mymensingh, a northern district in Bangladesh. Suspected rickettsial cases were enrolled from 2003 to 2005 on the basis of fever, rash and those patients who initially were diagnosed as typhoid and malaria cases but were nonresponsive to ciprofloxacin and anti-malarial therapy. Rickettsial diagnosis was confirmed by the Weil-Felix test [7].

Leptospirosis is a zoonotic infectious disease transmitted from animals to humans through contact of mucous membranes with water or soil contaminated with urine of infected animals [8]. Sixty-three leptospirosis patients were detected through serological evaluation in a study at two hospitals in Dhaka city in 2001 [9]. This study emphasized the need for further information on wide geographical context in Bangladesh.

Bacteria of the genus *Bartonella* cause human and animal disease and are transmitted between animals or is communicable to humans through diverse arthropod vectors (i.e. sand flies for *B. bacilliformis* or human body lice for *B. Quintana*) [10]. Many new *Bartonella* species have been identified in Indonesia, the Philippines, Singapore, and Thailand [10]. In addition, a study conducted in Dhaka, Bangladesh revealed a high prevalence of *Bartonella* in three mammalian species: lesser bandicoots, black rats, and house shrews [11]. Therefore, communities in Bangladesh could also be at risk of acquiring *Bartonella* infection.

Chikungunya is a viral disease, transmitted by *Aedes aegypti* and *Aedes albopictus* mosquitoes and manifested with incapacitating arthralgia [12]. In early 2006, the World Health Organization (WHO) reported a large Chikungunya outbreak in several Indian Ocean islands including the Maldives, Mauritius, Madagascar, Mayotte, Seychelles and La Reunion as well as in 151 districts located in ten states along the coastal region of India [12]. Dengue and Chikungunya viruses share the same vectors, similar symptoms and geographical distribution [13]; still we know little about the epidemiology of Chikungunya virus in Bangladesh [14].

While these studies have indicated the circulation of *Rickettsia, Leptospira, Bartonella* and Chikungunya virus in Bangladesh, they were often carried out in a limited setting and with a small sample size. It is also unknown how widespread these infections are and about their virulence for causing serious illnesses. We conducted a 12 month hospital-based study to determine the prevalence of various pathogens causing acute febrile illnesses in Bangladesh. We previously reported the 69 dengue fever and 4 malaria cases identified from this study [15]. This paper evaluates the distribution of *Rickettsia, Coxiella, Leptospira, Bartonella,* and Chikungunya virus among the febrile patients seeking hospital care in Bangladesh.

## Methods

### Study setting

The study setting, patient enrollment and sample collection method have been described previously [15]. The study period was from December 2008 to November 2009. Patients from inpatient and outpatient department of six tertiary teaching hospitals, from each division in Bangladesh, participated in this study.

### Case definition

We defined febrile cases as patients who presented with fever or a complaint of fever of >38 °C with onset within the preceding 10 days. We excluded any patient who developed a new onset of fever after 72 h of hospitalization. In addition, the study team excluded febrile patients with pneumonia, urinary tract infections, skin and soft tissue infections or a confirmed laboratory diagnosis of an infection other than the pathogens of interests, such as typhoid fever.

### Sample size and sampling procedure

We assumed that 200,000 people sought care for febrile illness at these hospitals per year, and if the real prevalence of infection with one of these organisms was 0.75%, then a sample of 675 would provide a 95% probability of identifying point prevalence between 0.1% and 1.4%. We aimed to collect 10 specimens from each hospital in each month [15]. This would provide 720 specimens in a year. Of these 10 specimens from each hospital, we collected 5 from the adult medicine unit and 5 from the pediatric unit (<14 years of age).

### Case enrollment

Two consecutive days in each month, field assistants and laboratory technicians from icddr,b (International Centre for Diarrheal Diseases Research, Bangladesh) visited the selected hospitals to assist the locally recruited study physicians. The study physicians first looked for inpatients who met the case definition. After obtaining consents/assents from the participants, the study physician enrolled up to 10 participants. If the study physician was unable to enrol samples from 10 inpatients then s/he collected the rest of the samples from outpatients using the same case definition.

### Data collection

The study physicians collected study-related demographic and clinical information in a standardized assessment form. Laboratory technicians attempted to follow-up by cell phone with all patients two months after their initial enrollment collecting information regarding recovery, residual illnesses and illnesses among the family members, locality, and select﻿﻿ed socio-economic variables.

### Specimen processing

Laboratory technicians inoculated Ellinghausen, McCullough, Johnson, and Harris (EMJH) medium with 3 drops of fresh whole human blood from the collected sample at the study hospital following sample collection. Thereafter, inoculated media were kept in an incubator at 30 °C at the icddr,b laboratory in Dhaka and shipped every two months for *Leptospira* laboratory analysis at the CDC. Laboratory technicians separated one third of the total blood in EDTA tube for real time polymerase chain reaction (PCR) for rickettsial DNA detection. The rest of the blood sample was dispensed into a sterile test tube which was centrifuged in the study location to get the blood cell concentrate and serum separated for *Bartonella* culture, enzyme-linked immuno sorbent assays (ELISA) and PCR tests. The blood cell concentrate was transferred into sterile 2 ml cryovials for *Bartonella* laboratory tests. Two aliquots of serum were preserved in screw capped cryovials: one for *Rickettsia* and *Coxiella* serological tests and one aliquot for Chikungunya virus serological tests.

### Laboratory tests

At the CDC, *Leptospira* species were isolated from the culture media. In addition, serovar classifications of isolated *Leptospira* species were performed by pulsed field gel electrophoresis (PFGE) [16]. For *Bartonella* testing, blood cell concentrate from each patient were inoculated into *Bartonella*/alpha-Proteobacteria growth medium (BAPGM) and incubated aerobically at 35 °C with 5% CO2 for seven days. DNA was extracted from this pre-enrichment using the QIAamp DNA mini kit (Qiagen, Chasworth, CA) according to manufacturer’s instructions, and analyzed using polymerase chain reaction (PCR) assays targeting specific regions in transfer-messenger RNA (ssrA), citrate synthase (gltA) gene, and 16S-23S rRNA internal transcribed spacer (ITS) [17]. Chikungunya laboratory tests were performed only for 99 patients who had both fever and joint pain. Chikungunya antibody was detected by ELISA and followed by a plaque reduction neutralization test (PRNT) [18]. PRNT was performed for four related alphaviruses, Ross River virus, Sindbis virus, Getah virus, and Bebaru virus; these viruses had previously been identified in Asia and thus were considered the most likely viruses to generate related antibodies. The immuno flourescence antibody test (IFAT) and PCR laboratory assays were also performed for all samples to detect spotted fever and typhus group *Rickettsia* [19, 20]. However, ELISA was performed for the 360 samples collected from December 2008 to May 2009 and IFAT for the remaining 360 samples collected from June to November 2009 for scrub typhus group *Rickettsia*. Samples collected from June to November 2009 were analysed for *Coxiella burnetii* specific antibody by ELISA assay.

### Diagnostic definitions

A patient was considered to be a case of leptospirosis if his/her blood culture of EMJH media grew *Leptospira*. Spotted fever, typhus group or scrub typhus group *Rickettsia* was diagnosed with a positive IFAT test at a titer >64 [21, 22] or for scrub typhus an ELISA positive test result. *Coxiella burnetii* was diagnosed by a positive ELISA test result. A febrile patient was diagnosed with bartonellosis by positive blood culture. Chikungunya fever was diagnosed by the presence of Chikungunya virus specific IgM or IgG antibody and PRNT with a titer ≥320 in patients presenting with both fever and joint pain.

### Data analysis

The primary outcome was the descriptive statistics of patients presenting with fever without confirmed laboratory diagnosis at six facilities considering the specific infections of interest. We performed principle component analysis based on 10 owned assets of the households, as well as sanitation facilities, drinking water sources, cooking fuels and household construction and divided this wealth index into 5 quintiles [23]; we presented the quintiles by our pathogens of interest.

### Ethical considerations

The study physicians obtained written consent from the adult participants and parents of children under 7 years of age and assents from children between 7 and 17 years old. The Ethical Review Committee (ERC) of icddr,b reviewed and approved the study protocol.

## Results

We enrolled 720 participants with a mean age of 19 years. Out of 462 inpatients, 232 were from the medicine unit; 147 (32%, *N* = 462) were female. Among 258 outpatients, 132 were from the medicine unit and 121 (47%, *N* = 258) were female. In total, 176 (24%) were under five years of age and 392 (54%) were less than 18 years of age.

Out of 720 febrile patients evaluated, 263 (37%) had laboratory results suggesting infection with one or more rickettsial agents (Table 1). Among 462 hospitalized patients, 150 (32%) had evidence of rickettsial infections. Ten patients (1%, *n* = 720) had evidence of rickettsial typhus group infection, 132 patients (18%, *n* = 720) for spotted fever, and 63 patients (18%, *n* = 360) had a scrub typhus fever IFA titer of more than 64 (Table 2).

**Table 1:** Distribution of pathogens among the febrile patients*

		ELISA screening [*N* = 360]†	IFA test (titer > 64) [*N* = 720]‡	PRNT (titer ≥ 320) [*N* = 99]	PCR [*N* = 720]§	Culture [*N* = 720]
Rickettsial infections	Typhus group (*N* = 720)	NT	10 (1)	NA	1 (0.1)	NT
	Spotted fever group (*N* = 720)	NT	132 (18)	NA	0	NT
	Scrub typhus group (*N* = 360)	NT	63 (18)	NA	2 (1)	NT
	Scrub typhus group (*N* = 360)	107 (30)	NT	NA	NT	NT
*Coxiella burnetii (N = 360)*		10 (3)	0	NA	0	NT
Leptospirosis (*N* = 720)		NT	NA	NA	NT	2 (0.3)
Chikungunya fever (*N* = 99)		10 (10)	NA	10 (10)	NT	NT
*Bartonella* infections (*N* = 720)		NT	NA	NA	NT	1 (0.1)

NT = not tested, NA = not applicable

* Values are n (%) unless otherwise indicated

† ELISA for Chikungunya virus were screened for 99 patients

‡ IFAT to detect *Coxiella* were performed for patients with positive or equivocal ELISA results

§ PCR for scrub typhus and *Coxiella* were performed for 360 patients

**Table 2:** Presentation of rickettsial species according to the titer of Indirect Immunofluorescence Assay (IFA) tests*

Species	32 titer	64 titer	128 titer	256 titer	512 titer or more
Typhus group (*N* = 720)	11 (2)	12 (2)	6 (1)	2 (0.3)	2 (0.3)
Spotted fever group (*N* = 720)	163 (23)	117 (16)	84 (12)	43 (6)	5 (1)
Scrub typhus group (*N* = 360)	13 (4)	5 (1)	14 (4)	15 (4)	34 (9)

* Values are n (%) unless otherwise indicated

**Fig. 1:**
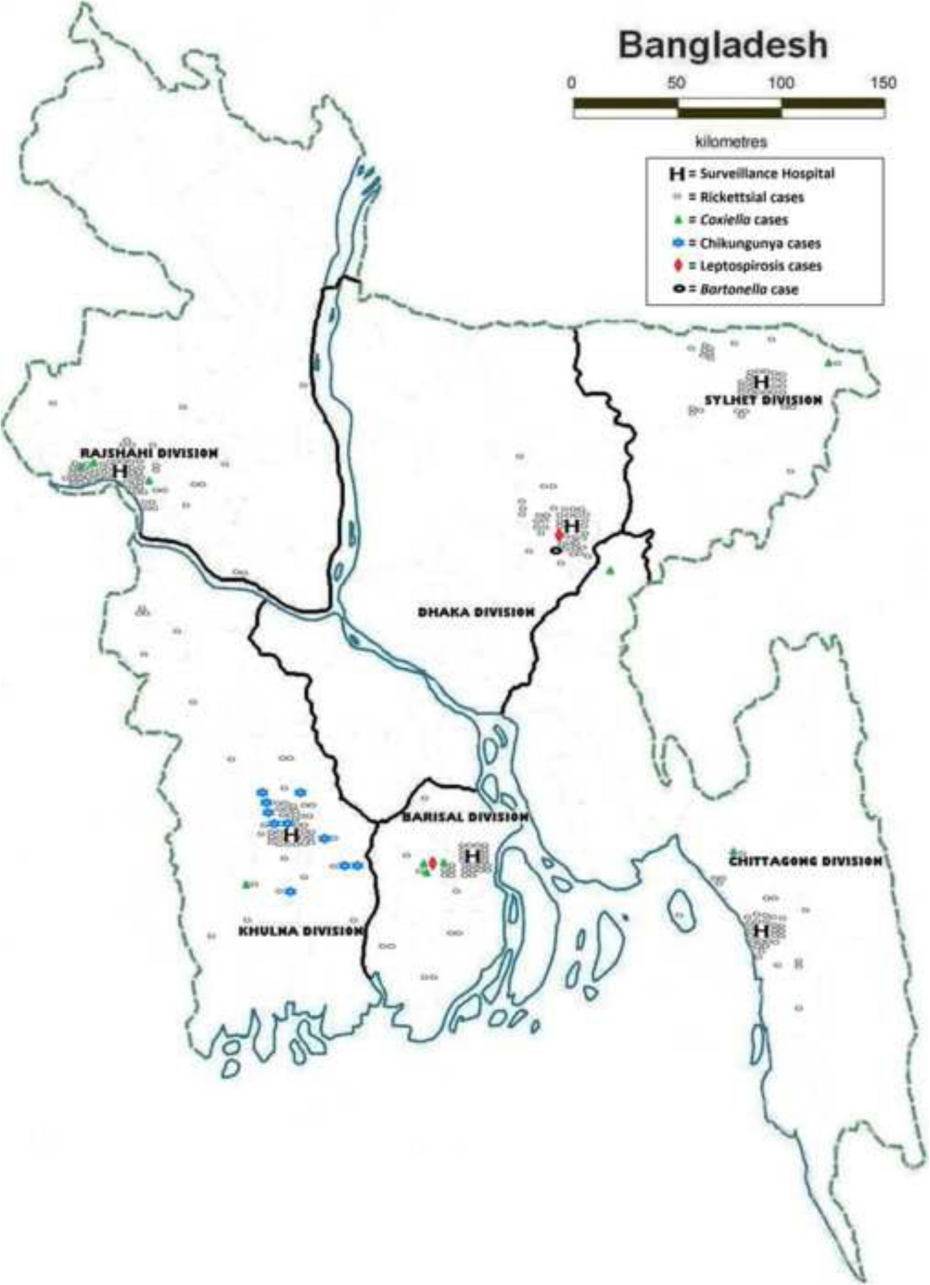
Map of Bangladesh showing the distribution of febrile patients with confirmed aetiologies

**Fig. 2:**
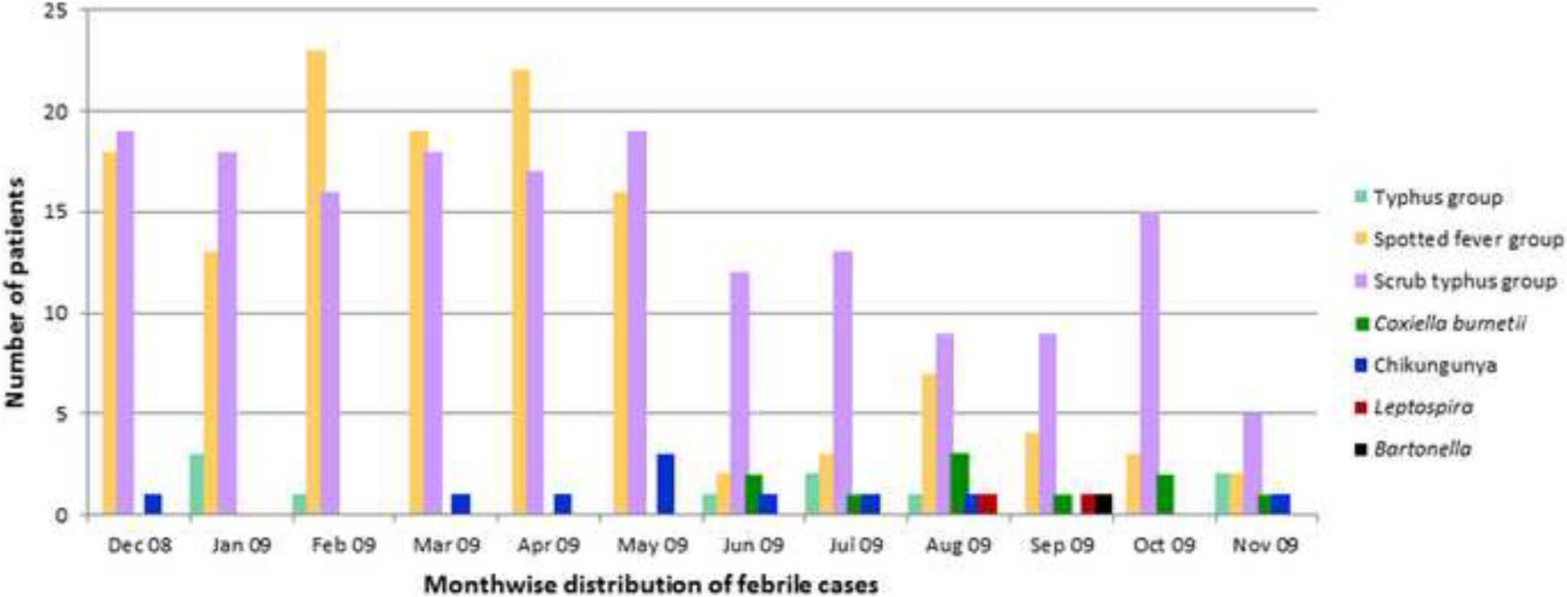
Presentation of pathogens among the febrile patients throughout the year from December 2008 to November 2009. * The laboratory tests for *Coxiella burnetii (N = 360)* were not performed for 360 samples collected from December 2008 to May 2009

Rickettsial illnesses were identified throughout Bangladesh, most commonly from patients tested from Rajshahi (*n* = 64, 53%) and least commonly from Barisal (*n* = 34, 28%) (Fig. 1). Evidence of typhus group, spotted fever group and scrub typhus group *Rickettsia* illnesses were detected throughout the year (Fig. 2). Among 34 patients having positive scrub typhus IFA titer >512, only two were diagnosed in November. Out of the 360 tested samples collected from June to November 2009, 10 were seropositive for *Coxiella*; 7 (70%) of these patients were from public hospitals and 6 (86%) of them were admitted to the inpatient unit. Three *Coxiella*-positive patients were identified from Rajshahi and Barisal equally and mostly in August (3) (Figs. 1 and 2).

Fever, headache, bodyache, muscle and joint pain were more frequent clinical presentations among the rickettsial patients. However, rash was confirmed on physical examination in only 2% of spotted fever group and 1% of scrub typhus group rickettsial illnesses.

**Table 3:** Characteristics of the study participants*

	*Rickettsia*† (*N* = 263)			*Coxiella burnetii* (*N* = 10)	*Leptospira* (*N* = 2)	Chikungunya (*N* = 10)	*Bartonella* (*N* = 1)
	Typhus group *Rickettsia* (*N* = 10)	Spotted fever group *Rickettsia* (*N* = 132)	Scrub typhus group *Rickettsia* (*N* = 170)				
Median (interquartile range) 14(5,30)	18(14,35)	17.5 (7,30)	30(12,45)	21.5(14,36)	24.5(7,42)	14(10,35)	9
Age category							
0-10 years (318)	2 (1)	50 (16)	39 (12)	2 (1)	1 (0.3)	3 (1)	1 (0.3)
11-20 years (121)	4 (3)	24 (20)	24 (20)	3 (2)	0	3 (2)	0
21-30 years (117)	1 (1)	26 (22)	28 (24)	1 (1)	0	0	0
31-40 years (62)	2 (3)	17 (27)	30 (48)	2 (3)	0	4 (6)	0
> 40 years (102)	1 (1)	15 (15)	49 (48)	2 (2)	1 (1)	0	0
Below 5 years (176)	1 (1)	28 (16)	13 (7)	1 (1)	0	1 (1)	0
Below 18 years (392)	4 (1)	66 (17)	55 (14)	4 (1)	1 (0.3)	6 (2)	1 (0.3)
Male (452)	7 (2)	76 (17)	90 (20)	6 (1)	2 (0.4)	4 (1)	1 (0.2)
Inpatient medicine (232)	5 (2)	29 (13)	72 (31)	7 (3)	1 (0.4)	3 (1)	0
Inpatient paediatrics (230)	1 (0.4)	32 (14)	34 (15)	2 (1)	1 (0.4)	4 (2)	0
Wealth index‡ [*N* = 557]§	(*N* = 5)	(*N* = 94)	(*N* = 130)	(*N* = 8)	(*N* = 1)	(*N* = 6)	(*N* = 1)
Lowest (110)	1 (1)	18 (16)	32 (29)	2 (2)	0	0	0
Second (110)	0	22 (20)	37 (34)	2 (2)	1 (1)	3 (3)	0
Middle (106)	0	17 (16)	18 (17)	2 (2)	0	2 (2)	1 (1)
Fourth (114)	1 (1)	18 (16)	24 (21)	2 (2)	0	0	0
Highest (117)	3 (3)	19 (16)	19 (16)	0	0	1 (1)	0

* Values are n (%) unless otherwise indicated; Mean is reported along with ± Standard deviation

† *Rickettsia* test results categorized as positive cases who had IFAT titer of more than 64 or scrub typhus ELISA positive. Rickettsial species included typhus group, spotted fever group and scrub typhus group*.* In total, 312 rickettsial infections (typhus group [*n* = 10], spotted fever group [*n* = 132] and scrub typhus group [*n* = 170]) identified in 263 patients

‡ Quintile 1 to 5 of wealth index is based on principle component analysis on the owned 10 assets of the households, sanitation facilities, drinking water sources, cooking fuels and household construction

§ We could follow up in total 557 out of 720 febrile patients; 5 out of 10 typhus group, 94 out of 132 spotted fever group, 130 out of 170 scrub typhus group, 8 out of 10 *Coxiella burnetii* cases, 1 out of 2 leptospirosis, 6 out of 10 Chikungunya and one bartonellosis patients

Among two identified leptospirosis cases, one was a 42 year-old man from Kishoreganj with a history of intermittent fever and headache and a neutrophil count of 86%. The other case was a 7 year-old boy from Barisal with history of continuous fever, signs of jaundice and a lymphocyte count of 41% (Table 3). The *Leptospira* isolate from Barisal was *L. borgpetersenii* serovar Ceylonica, a member of serogroup Javanica, with a similar PFGE pattern to serovars Dehong, 52-73, and 27-75. The second *Leptospira* isolate was of serogroup Sejroe and by PFGE it was *L. borgpetersenii* serovar Dikkeni.

One 9 year-old male outpatient from Kishoreganj presented with a one day history of continuous fever without rash or travel history with concomitant diagnosis of dengue fever (15) and *Bartonella* infection (Table 3). The blood sample enriched in liquid BAPGM medium was PCR positive for *Bartonella* with all three targets (*gltA*, *ssrA,* and ITS) and sequencing analyses of *ssrA* and ITS demonstrated that it belonged to *Bartonella elizabethae*, a rat-associated *Bartonella* species.

Ninety-nine samples from people who had a history of both fever and joint pain were tested for antibodies against Chikungunya virus. Ten samples were positive using IgG ELISA tests; one was positive to both IgG and IgM. The IgG ELISA results were confirmed by PRNT results with all titers on positive samples greater than or equal to 320. In addition, all PRNT results were negative for 4 related alphaviruses.

The case fatality rate among patients with rickettsial infections was 5% (*n* = 10, *N* = 191) (Table 4). The case fatality rate for patients without a diagnosis of *Rickettsia* was 2% (*n* = 7, *N* = 357). Among the patients who died with evidence suggesting a *Rickettsia* infection, five patients had scrub typhus titers, three were spotted fever positive, and two had positive test results for both agents. Co-infection appeared higher among spotted fever and scrub typhus group positive patients with a proportion of 6% (*n* = 45, *N* = 720). Among these, five patients (11%, *n* = 5, *N* = 45) were co-infected with either dengue or Chikungunya viruses (Table 5). Febrile patients commonly presented with intermittent fever. Fever was associated with chills or sweating and subsided either without taking any medication or with only antipyretic medication such as paracetamol (Table 6).

**Table 4:** Clinical presentation and outcome*

	*Rickettsia* (*N* = 263)			*Coxiella burnetii* (*N* = 10)	*Leptospira* (*N* = 2)	Chikungunya (*N* = 10)	*Bartonella* (*N* = 1)
	Typhus group *Rickettsia* (*N* = 10)	Spotted fever group *Rickettsia* (*N* = 132)	Scrub typhus group *Rickettsia* (*N* = 170)				
Symptoms							
Fever	10 (100)	132 (100)	170 (100)	10 (100)	2 (100)	10 (100)	1 (100)
Headache	7 (70)	66 (50)	117 (69)	5 (50)	1 (50)	3 (30)	0
Bodyache	3 (30)	54 (41)	84 (49)	4 (40)	0	2 (20)	0
Muscle pain	0	23 (17)	38 (22)	0	0	0	0
Joint pain	0	19 (14)	46 (27)	0	0	10 (100)	0
Rash	0	1 (1)	2 (1)	0	0	0	0
Bleeding	0	2 (2)	2 (1)	0	0	0	0
Retro-orbital pain	0	5 (4)	13 (8)	0	0	0	0
Eye-redness	0	5 (4)	5 (3)	0	0	0	0
Jaundice	0	4 (3)	8 (5)	2 (20)	0	0	0
Neck stiffness	0	2 (2)	4 (2)	0	0	0	0
Low urinary output	0	5 (4)	3 (2)	0	0	1 (10)	0
Physical findings							
Mean Pulse rate	101 ± 19	100 ± 21	92 ± 21	96 ± 19	100 ± 0	95 ± 19	130 ± 0
Mean Respiratory rate	20 ± 6	26 ± 12	23 ± 11	22 ± 9	28 ± 3	26 ± 7	26 ± 0
Mean Systolic BP	115 ± 16	108 ± 17	110 ± 19	101 ± 12	110 ± 14	103 ± 11	110 ± 0
Mean Diastolic BP	74 ± 8	71 ± 10	71 ± 12	63 ± 9	60 ± 0	68 ± 9	60 ± 0
Mean Temperature	99.7 ± 1.5	100.2 ± 1.7	100.1 ± 1.5	100.1 ± 1.3	102.5 ± 2.1	99.8 ± 0.6	99 ± 0
Anemia	1 (10)	21 (16)	34 (20)	3 (30)	0	2 (20)	0
Jaundice	0	3 (2)	6 (4)	1 (10)	1 (50)	0	0
Edema	0	4 (3)	6 (4)	0	0	0	0
Rash	0	2 (2)	2 (1)	0	0	0	0
Neck rigidity	0	2 (2)	5 (3)	0	0	0	0
Dehydration	0	13 (10)	23 (14)	2 (20)	0	0	0
Laboratory findings							
Mean WBC count	8000 ± 1732	12656 ± 9544	10570 ± 3148	11225 ± 6352	10850 ± 3889	-	-
Mean Neutrophil percent	63 ± 8	68 ± 14	66 ± 15	67 ± 12	71 ± 21	-	-
Mean Platelet count	-	228733 ± 132686	279455 ± 136035	226500 ± 37477	185000 ± 0	-	-
Mean ESR	42 ± 20	40 ± 20	48 ± 29	34 ± 10	58 ± 3	28 ± 0	-
Outcome [N = 557]†	(*N* = 5)	(*N* = 94)	(*N* = 130)	(*N* = 8)	(*N* = 1)	(*N* = 6)	(*N* = 1)
Recovery‡	5 (100)	59 (63)	76 (58)	6 (75)	1 (100)	3 (50)	1 (100)
Death	0	5 (5)	7 (5)	2 (25)	0	0	0
Mean recovery days§	11 ± 7	17 ± 12	18 ± 10	16 ± 12	18 ± 0	28 ± 9	16 ± 0
Similar Concurrent illness in the family member	0	11 (12)	8 (6)	0	0	2 (33)	0

* Values are n (%) unless otherwise indicated; Mean is reported along with ± Standard deviation

† We could follow up 5 out of 10 typhus group, 94 out of 132 spotted fever, 130 out of 170 scrub typhus group, 8 out of 10 *Coxiella burnetii* cases and 1 out of 2 leptospirosis, 6 out of 10 Chikungunya and one bartonellosis patients. The remaining patients were not accessible or available on phone follow-up

‡ Recovery is defined as presence of no signs or symptoms of illness or any disability

§ The mean recovery days along with SD indicates how long it took time for the febrile patients to recover from the illnesses

**Table 5:** Co-seropositivity of dengue, malaria, Chikungunya with rickettsial illnesses *and Coxiella burnetii*

			*n* (%)
Any two rickettsial illnesses	Typhus group	Spotted fever	3 (0.4)
	Spotted fever	Scrub typhus	45 (6)
	Typhus group	Scrub typhus	1 (0.1)
*Coxiella burnetii* with any two rickettsial illnesses	Typhus group	Scrub typhus	1 (0.1)
	Spotted fever	Scrub typhus	1 (0.1)
*Coxiella burnetii* with any one rickettsial illnesses	Scrub typhus		3 (0.4)
Chikungunya with any two rickettsial illnesses	Spotted fever	Scrub typhus	5 (1)
	Scrub typhus		1 (0.1)
Dengue with any two rickettsial illnesses	Spotted fever	Scrub typhus	5 (1)
Dengue with any one rickettsial illnesses	Typhus group		1 (0.1)
	Spotted fever		9 (1)
	Scrub typhus		10 (1)
Dengue	*Coxiella burnetii*		1 (0.1)
Malaria (*P. falciparum*)	*Coxiella burnetii*		1 (0.1)

**Table 6:** Pattern of fever among the febrile patients*

	Typhus group *Rickettsia* (*N* = 10)	Spotted fever group *Rickettsia* (*N* = 132)	Scrub typhus group *Rickettsia* (*N* = 170)	*C. burnetii* (*N* = 10)	*Leptospira* (*N* = 2)	Chikungunya (*N* = 10)	*Bartonella* (*N* = 1)
Fever type:†							
Remittent	2 (20)	23 (17)	25 (15)	0	0	0	0
Intermittent	8 (80)	72 (55)	93 (55)	9 (90)	1 (50)	7 (70)	0
Continuous	0	33 (25)	44 (26)	1 (10)	1 (50)	3 (30)	1 (100)
Fever subsided with:							
Paracetamol	3 (30)	81 (61)	113 (66)	4 (40)	0	8 (80)	0
Analgesics	0	1 (1)	2 (1)	0	0	1 (10)	0
Both	0	0	0	0	0	0	0
No medication	7 (70)	50 (38)	55 (32)	6 (60)	2 (100)	1 (10)	1 (100)
Fever associated with:							
Chills	0	12 (9)	22 (13)	1 (10)	1 (50)	2 (20)	0
Sweating	0	17 (13)	22 (13)	2 (20)	0	6 (60)	0
Both	0	2 (2)	5 (3)	0	0	0	0

* Values are n (%) unless otherwise indicated

† Fever type: Intermittent fever presents with elevated temperature but falls to normal (37 .2 °C or below) each day and in case of remittent fever, the temperature falls each day but not comes to normal. However, in continuous fever, there is little change (0 .3 °C or less) in the elevated temperature during a 24-h period

Out of 720 participants, 462 patients were from inpatients so the classifications were mostly based on objective assessment while hospitalized; however 258 outpatients reported on their febrile history

Out of 187 patients later identified with *Rickettsia* from our laboratory testing, 49 (26%) febrile patients were initially diagnosed clinically with enteric fever, 26 (14%) patients with respiratory tract infection (RTI), 14 (7%) patients with viral fever, and 12 (6%) with malaria on admission. Among the 10 patients who died and were positive for rickettsial infection, two were diagnosed with malaria, one with meningo-encephalitis, and one with hepatitis. The remaining five patients were diagnosed with either fever with acute liver failure, febrile convulsion, cerebral palsy with bronchopneumonia, cerebrovascular disease (CVD) with renal impairment or bronchogenic carcinoma and no diagnosis was reported for one patient. Out of 242 rickettsial-positive patients, 41 (17%) were treated exclusively with ceftriaxone, 34 (14%) with azithromycin alone, 22 (9%) with ciprofloxacin only, 21 (9%) with amoxicillin, and 13 (5%) were treated with levofloxacin. Doxycycline was prescribed in only one patient and tetracycline in two patients. Out of ten deaths, an antibiotic was prescribed for only six of them; three of them were prescribed paracetamol and one ibuprofen.

## Discussion

Rickettsial disease is widespread in Bangladesh. Patients with evidence of a rickettsial infection had a higher case fatality ratio compared to the febrile patients with other infectious aetiologies. Other causes of acute febrile illness such as leptospirosis (0.3%), bartonellosis (0.1%) and Chikungunya fever with joint pain (10%) were also identified from Bangladesh.

An earlier study on rickettsial disease in Bangladesh was conducted at Mymensingh Medical College Hospital where 40 rickettsial cases were described including 48% with scrub typhus and 40% with Indian tick typhus, a member of the spotted fever group. However, this was a small study with patients limited to only one district, and only selected rickettsia-positive cases were evaluated without any routine surveillance or standard case definition. In addition, the Weil–Felix agglutination reaction test that was used for diagnosis of rickettsial cases, has low sensitivity and specificity. Moreover, this test was performed in various local laboratories not at reference laboratories [7].

A high titer for scrub typhus suggests active/recent infection and is likely the cause of the febrile illness in the patients diagnosed with rickettsial fever (Table 2). Low titers are more indicative of a past exposure although the titer will also be low during the early stage of an infection. The results presented here are most consistent with active scrub typhus infections at the time of the febrile illness [24, 25]. The temporal distribution suggests that scrub typhus infections occur throughout the year; however, this might be due to the detection of active infections in the rainy seasons and detection of past rainy-season infections during the winter months. Active infections had been generally reported during the summer rainy season when the trombiculid mites are most abundant and active [26–30]. Similarly, the year-round distribution of typhus group and spotted fever group [31–33] presenting with a lower titer suggests past exposure for most of these rickettsial cases (Fig. 2); the proportion of patients identified with typhus group and having an IFA titer below 512 was 80% and for spotted fever group 96%, and for an IFA titer at 128 was 60% for typhus group and 64% for spotted fever group. The high proportion of low titers (i.e., 32 and 64) in both these groups suggests that these are not active infections but might be explained by prior exposure to infections among these febrile patients (Table 2).

The case fatality rate of rickettsial fever has been reported to be up to 30-35% if untreated [25, 34], the age adjusted Rocky Mountain spotted fever (RMSF) case fatality rate ranged from 10 to 25% [35], and even with treatment, 5% of the cases died [36]. Likewise, scrub typhus mortality of 10–50% has been reported in untreated patients [37]. This higher mortality is often due to delayed consultation with physicians or failure to prescribe appropriate antibiotics [25, 34, 38]. In addition, the diagnosis could be difficult due to absence of rash or eschar or under-reporting of antecedent arthropod vector bite [35]; the clinical parameters in our study did not support separating the rickettsial cases based on any clinical criteria. Similarly, we observed higher case fatality ratio among patients suffering from rickettsial fever compared to other febrile illnesses in this study. Rickettsial diagnoses were missed in these hospitals likely due to low index of clinical suspicion and inadequate diagnostic facilities. Only one rickettsial patient in this study was treated with doxycycline, the drug of choice for rickettsial infection of both adults and children [19].

Earlier studies in urban [9, 39] and rural [40] Bangladesh identified *Leptospira* infections. Eight percent probable and definitive leptospirosis cases were detected through microscopic agglutination tests (MAT) on 584 febrile patients from Kamalapur, a low income area in Dhaka city. On the other hand, 33% of 58 febrile cases with/without jaundice were diagnosed as leptospirosis patients using a microcapsule agglutination test (MCAT) from Sirajganj, a rural district in Bangladesh. Additionally, in a hospital-based study, 18% of 359 dengue negative serum samples were confirmed as PCR positive leptospirosis cases from Dhaka Medical College Hospital and Holy Family Red Crescent Hospital in 2001 [9]. However, we confirmed only two leptospirosis cases using EMJH culture media throughout Bangladesh for a period of one year. The earlier studies were based on highly sensitive serological tests [41–43], so our culture-based approach underestimated the contribution of *Leptospira* to the burden of febrile illness in these hospitals.

We identified *Leptospira borgpetersenii* serovar Ceylonica (serogroup Javanica) and *L. borgpetersenii* serovar Dikkeni (serogroup Sejroe) in this study. These serovars were originally isolated in nearby geographic regions [44–47].

A zoonotic study in 2003 in Bangladesh reported that 43% of 201 mammals were infected with *Bartonella* and most of the identified organisms resembled *B. elizabethae* [11]. We confirmed one *Bartonella* (*B. elizabethae*) case by culture in this study. Although our culture based approach allowed us to characterize the isolate, it almost certainly underestimated the contribution of *Bartonella* to febrile illness in these hospitals [48].

A study was conducted by IEDCR and icddr,b to identify Chikungunya fever in urban Dhaka in 2006 with 175 blood samples [49]. The study site was limited to Dhaka, the sample size was small and did not identify any evidence of Chikungunya virus in Bangladesh [49]. During our study period in December 2008, the first Chikungunya outbreak had been reported from Rajshahi and Chapianawabganj districts of Bangladesh [14]. However, we identified Chikungunya cases throughout the year from Khulna, a district in southern Bangladesh, suggesting Chikungunya fever was circulating in Bangladesh and in neighbouring states of India where re-emergence of this virus had already been reported [49].

Our study had some limitations. This was a hospital based study, and therefore excluded acute febrile patients who never visited these hospitals. However, the selected study sites were the regional referral hospitals and had a wide catchment area. In addition, the selected pathogens often result in serious febrile and other clinical presentations which require hospitalizations or hospital visits. Therefore, the study hospitals were likely able to capture most of the febrile-illness causing pathogens that were targeted.

Secondly, all samples were not tested for all pathogens. Only samples from febrile patients with joint pain were tested for Chikungunya virus and only samples collected from June to November 2009 were tested for *Coxiella burnetii*. However, earlier studies demonstrate that joint pain is an obligatory feature for Chikungunya fever [12] and *C. burnetii* has been commonly reported in early spring and summer months [6, 50].

Thirdly, the methods used for rickettsial diagnosis were primarily serological tests. There were no paired samples to evaluate the changes of titres for rickettsial serological laboratory analysis. However, a single IFA titre of > 64 which has been used as a cut-off for the diagnosis of rickettsia infection in this study, would likely to confirm the presence of rickettsia infection in Bangladesh. Some of these positive serological tests were likely from prior infection, and might not be the cause of the presenting febrile illness including those illnesses leading to death. Although this testing strategy may have overestimated active rickettsial infections, the high IFAT titers detected in many patients during the study period were strongly suggestive of acute infections. On the other hand, the culture-based approach likely underestimated the prevalence of leptospirosis and bartonellosis. However, these culture methods were important for confirmation and identification of specific *Bartonella* or *Leptospira* species.

## Conclusions

Rickettsial diseases are a common cause of febrile illness in Bangladesh. These febrile patients are either left undiagnosed or misdiagnosed (for example, enteric fever). The feasibility of using more expensive reliable rickettsial diagnostics such as IFAT (USD 24.60 per test) or PCR (cost variable) tests in the local context needs to be weighed against the alternative of more widespread prescription of available inexpensive antibiotics. Although the low cost of the Weil-Felix test makes it attractive to laboratories in Bangladesh, the low sensitivity and specificity means that it provides limited sound guidance to clinical decisions. Population based studies would help identify the local environmental and socio-cultural factors behind these identified infections to adopt appropriate interventions. Clinical management guidelines for febrile patients should be revised considering other prevailing causes of febrile illness in the country in addition to malaria and dengue.

## Abbreviations

BAPGM: Bartonella/alpha-proteobacteria growth medium
CDC: Centers for Disease Control and Prevention
CVD: Cerebrovascular disease
EDTA: Ethylenediaminetetraacetic acid
ELISA: Enzyme-linked immuno sorbent assays
EMJH: Ellinghausen, McCullough, Johnson, and Harris medium
ERC: Ethical review committee
icddr,b: International Centre for Diarrheal Diseases Research, Bangladesh
IEDCR: Institute of Epidemiology, Disease Control and Research
IFAT: Immuno flourescence antibody test
ITS: Internal transcribed spacer
MAT: Microscopic agglutination tests
MCAT: Microcapsule agglutination test
PCR: Polymerase chain reaction
PFGE: Pulsed field gel electrophoresis
PRNT: Plaque reduction neutralization test
RMSF: Rocky Mountain spotted fever
RTI: Respiratory tract infection
USD: United States dollar
WHO: World Health Organization
